# Connecting and integrating cooperation within and between species

**DOI:** 10.1098/rstb.2023.0203

**Published:** 2024-07-22

**Authors:** Judith L. Bronstein, Hari Sridhar

**Affiliations:** ^1^ Department of Ecology and Evolutionary Biology, University of Arizona, Tucson, AZ 85721, USA; ^2^ Konrad Lorenz Institute for Evolution and Cognition Research, Klosterneuburg A-3400, Austria

**Keywords:** mutualism, mixed-species group, animal sociality, interspecific interaction, positive interaction, cooperation

## Abstract

There has long been a fundamental divide in the study of cooperation: researchers focus either on cooperation within species, including but not limited to sociality, or else on cooperation between species, commonly termed mutualism. Here, we explore the ecologically and evolutionarily significant ways in which within- and between-species cooperation interact. We highlight two primary cross-linkages. First, cooperation of one type can change the context in which cooperation of the other type functions, and thus potentially its outcome. We delineate three possibilities: (i) within-species cooperation modulates benefits for a heterospecific partner; (ii) between-species cooperation affects the dynamics of within-species cooperation; and (iii) both processes take place interactively. The second type of cross-linkage emerges when resources or services that cooperation makes available are obtainable either from members of the same species or from different species. This brings cooperation at the two levels into direct interaction, to some extent obscuring the distinction between them. We expand on these intersections between within- and between-species cooperation in a diversity of taxa and interaction types. These interactions have the potential to weave together social networks and trophic dynamics, contributing to the structure and functioning of ecological communities in ways that are just beginning to be explored.

This article is part of the theme issue ‘Connected interactions: enriching food web research by spatial and social interactions’.

## 1. Introduction

The study of cooperation has flourished in recent years. A large body of research has accumulated that addresses key questions about its ecology and evolution, notably, when cooperation will arise and conditions under which it can persist in the face of competition and the so-called temptation to cheat. The study of cooperation subsumes nearly all taxa, from microbes to humans occupying habitats worldwide, and focuses on time scales ranging from seconds to millennia. It is observational, experimental and theoretical. No single body of theory is yet able to integrate all this knowledge, but advances that cut across specific systems are accumulating rapidly.

There has long been one fundamental divide in the study of cooperation: a focus on cooperation within species, including but not limited to sociality, versus a focus on cooperation between different species (see §2 for a discussion of terminology). There has been little integration, and indeed limited exchange, among researchers working on cooperation at these two levels ([1], but see e.g. [2–8]). Between-species cooperation (mutualism) is usually discussed in relation to other kinds of between-species interactions (e.g. predation, parasitism and commensalism). In turn, the within-species literature generally contrasts cooperation with less beneficent outcomes of the same interaction, notably competition.

The independent development of these bodies of knowledge has obscured two touch points between cooperation at the within- and between-species levels. First, it is clear that, in many ways, these two fields address the same puzzle: how can mutual benefit arise and persist when, in many cases, antagonistic behaviours rather than cooperation should be favoured? We still know surprisingly little about whether there are common answers to this question (but see e.g. [4,7,9]). Second, cooperation at the within- and between-species levels is not independent, but rather is entangled in ways that we are just beginning to consider. In this article, we explore this second touch point.

We argue here that there are two primary ways in which within- and between-species cooperation intersect. First, cooperation of one type can change the context in which cooperation of the other type functions, and thus potentially its outcome. Second, in some cases, resources or services that cooperation makes available can be obtained either from members of the same or different species, bringing cooperation at the two levels into direct interaction. Documenting how cooperation functions simultaneously at multiple levels is critical, we argue, for achieving an understanding of how social and trophic processes interact to affect the dynamics of networks of co-existing species.

**Figure 1.**
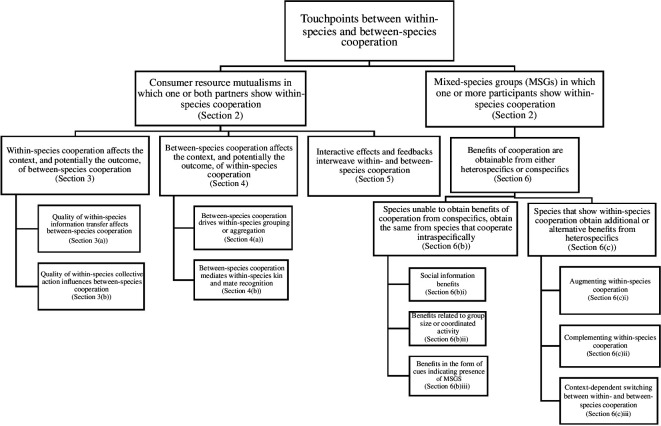
A roadmap of the conceptual framework developed in this article to understand intersections between within- and between-species cooperation. For further explanation and relevant examples go to the section IDs provided in the boxes.

Below, we first establish the terminology used in this article. We then briefly review the benefits that between- and within-species cooperation confer and from whom they can be obtained. The nature of these commodities of exchange, we argue, will fundamentally shape how cooperation at the two levels can interact. We then expand on each of these intersections between within- and between-species cooperation, highlighting our current understanding of a wide diversity of taxa and interaction types. Figure 1 provides an overview of the article’s organization and its key conceptual points. Our goal is to explore previously little-appreciated patterns and to suggest directions for further exploration, which we outline at the conclusion of the article (§7).

## 2. Forms of cooperation

Cooperation has been defined in many ways [10]. By one definition, it is the simultaneous or consecutive acting of two or more individuals by the same or different behaviours to achieve a shared goal [11]; by another, it is a behaviour that provides a benefit to another individual (the recipient) and that is selected for because of its beneficial effect on that recipient [10]. Note that these definitions (two of many, we note) do not specify whether these individuals belong to the same or different species. In this article, we focus on this distinction. What we will term *within-species cooperation* involves two or more individuals of the same species. Species that exhibit such behaviours often but not always live in social groups; by definition, they all take place at a single trophic level. We contrast within-species cooperation with *between-species cooperation*: interactions that benefit two or more species at the same time. In the ecological literature, such interactions are called *mutualisms*, a term we will use synonymously with between-species cooperation. The best-known mutualisms involve species at different trophic levels. However, as we discuss here, there are some that take place among species at the same trophic level.

There are key similarities between within- and between-species cooperation, as well as fundamental distinctions between them. These are important to recognize at the outset. To briefly summarize (for more detailed treatments, see e.g. [6]), the advantage of any form of cooperation is that it makes available benefits that are difficult or impossible to obtain independently. Hence, individuals fare better together than they would on their own. Cooperation is the outcome of a diverse set of traits in participating entities, ranging from behavioural to morphological, some simple and shared broadly across individuals, others unique and tuned to a specific partner or setting. The benefits of any type of cooperation accrue via increased survival and/or reproduction. Cooperation can be obligate or facultative, directed at specific partners or generalized in its expression. Cooperation of any type is an exchange, and as such, it is generally necessary to make investments to obtain benefits from another individual (but see [12] and [13]). Therefore, cooperation, as a rule, involves costs. Consequently, the opportunity arises to obtain benefits from others without providing any in return. As a consequence, failing to cooperate is seen as a temptation, and responses to individuals who do not cooperate are considered critical to the long-term persistence of cooperation [4].

Selection of the best cooperative partner—the best species, as well as the best specific individuals—is fundamentally linked to the benefits gained via cooperation. Cooperation associated with reproduction is generally a within-species phenomenon (with some exceptions; e.g. [14]). The most obvious example is cooperation among adults (often but not always mates) for the care of offspring: this benefit is almost exclusively accrued by cooperating with a conspecific. Cases in which group success is achieved via suppression of individual reproductive output, such as is seen in the reproductive division of labour in eusocial insects [15], are similarly within-species phenomena.

Direct reproductive benefits are not the only advantages accruing from cooperation, however. In many cases, cooperation is based on the exchange of resources, often termed either commodities or rewards and services. In these cases, individuals exchange commodities that they have in excess, for commodities that they require but either have no access to or cannot obtain affordably. These exchanges take place within a ‘biological marketplace’, with specific conditions predicting when the exchanges will be mutually beneficial versus exploitative [16]. Beneficial exchanges are not necessarily symmetrical: asymmetries might result, for instance, from differences in status or reputation [17,18]. Generally, the key to these exchanges are differences between partners in the commodities that they are able to produce in excess for trade. Hence, these interactions commonly involve different species. For example, plants synthesise nutritious rewards (usually nectar) for pollinating animals and receive in return the benefit of having pollen moved between flowers. Plants gain the benefit of directed movement—something difficult to obtain without the aid of a mobile species—while pollinators receive critical food resources.

A key point is that many of these heterospecific cooperative exchanges integrate not only different species but different trophic levels: they involve one species that is a producer (in the case above, the plant) and one that is a consumer (the pollinator). Consequently, mutualistic cooperation is in some ways more similar to other consumer–resource interactions, including predation and parasitism, than it is to within-species cooperation [19]. The difference is that in cooperative consumer–resource interactions, the species providing a resource benefits from the act of consumption, generally because what is consumed is a product produced by the partner rather than the partner itself (with some exceptions; e.g. [20]).

There are certain benefits that can be obtained by cooperating with a member of *either* the same or a different species. Individuals can partner with others to acquire food, for example. Group hunting usually involves members of the same species, but it can also be a heterospecific activity [21,22]. Predator vigilance, discussed in detail in §6, is another group behaviour that can involve members of the same or different species. In these cases, the different species involved in cooperation commonly occupy the same trophic level [23].

Moreover, in this latter class of interactions, cooperation for a given benefit (e.g. hunting, predator vigilance) can involve groups made up of members of the same and different species at the same time. These are most often termed *mixed-species groups* (MSGs) in the published literature, and we adopt this terminology here. In many (although not all) MSGs, members of the same or different species obtain identical benefit or benefits. In other cases that we will discuss, the benefits accrued by cooperating with members of the same species are different from the benefits that might be obtained from other species.

In the following sections, we delineate two general ways in which within- and between-species cooperation are interwoven (figure 1), leading to the integration of within- and between-trophic level dynamics.

## 3. Within-species cooperation affects between-species cooperation

Species that exhibit within-species cooperation commonly cooperate with other species as well. For example, social insects are prominent pollinators worldwide; ants protect and in turn benefit from clonal, colonial and parent–offspring groups of other insects; and social organisms collectively gather and manage individuals of other species to their mutual benefit. In all of these cases (discussed below), cooperative behaviour within and between species is not independent: rather, within-species cooperation in one species alters its cooperative interactions with a second species. Here, we focus specifically on how the outcome of between-species cooperation is linked to the social behaviour of one of the interacting species. We note that some cases we discuss involve a mutualism between a social species and a solitary one; others involve a mutualism between two species that both exhibit within-species cooperation. While this is an important distinction, and one we return to in §5, here our focus is on the outcome of the mutualism itself.

We can delineate two mechanisms by which between-species cooperation is likely to emerge and persist when at least one of the species is a within-species cooperator. First, between-species cooperation is facilitated when accurate and high-quality information about an associated species (e.g. its location, quantity or quality) can be shared among conspecifics. We term this *information transfer*. Second, between-species cooperation is facilitated when the location, collection and/or management of the associated species or the resource it provides can only be accomplished by a cooperative group. We term this *management benefits*. Below, we consider each of these mechanisms, in turn. We also highlight instances in which the within-species cooperator alone, rather than both species, might benefit from its actions.

### (a) Information transfer

As discussed in §2, mutualisms commonly take place between organisms at different trophic levels; benefits are received when one of these species (commonly termed a ‘partner’) consumes a valuable resource (commodity) produced by the other, and this consumption rebounds to the producer’s benefit. However, partners producing these commodities can be difficult to locate and/or the commodity itself difficult to collect. An advantage of within-species cooperation is that conspecifics can obtain knowledge from each other that aids them in succeeding at these tasks. Socially mediated improvement in the location and handling of commodities should in turn have a direct effect on the partner species, and on their mutualism itself.

Eusocial insects are probably the most widespread mutualists in terrestrial habitats worldwide. They are the most common pollinators [24] and occasional seed dispersers [25]; further, they commonly defend, in exchange for food, certain plants and insects from enemies [26,27]. Sharing and transfer of information are key to their effectiveness as partners in these interactions: as we discuss below, an array of mechanisms have evolved that allow them to locate food rapidly and then convey information about it to other colony members. As a consequence, they are able to rapidly recruit and exploit newly located food resources.

The sharing of information about mutualistic commodities is well documented among eusocial pollinators and defenders. Through the well-known ‘waggle dance’, for example, a honeybee forager informs her nestmates of the location and quality of nectar resources, allowing the colony to rapidly find and exploit food [28,29]. There are other modes of communication among bees as well. Trophallaxis, or direct food transfer between individuals, is performed in the nest by honey bees and stingless bees; it allows nestmates to learn the odours of available food sources [30]. Some bees also scent-mark flowers to signal that they have already visited them. An initial attraction towards the familiar scent of conspecifics may be reinforced when individuals learn to associate the marks with high nectar availability, or reversed when scent marks indicate depleted flowers [31].

Ant scouts communicate food locations to colony members by laying down pheromone trails [32]. These trails facilitate quick establishment of prolonged association between ant colonies and stable food resources offered by their partners, including plants that exchange protection for extrafloral nectar [33] and insects (including lycaenid butterfly larvae and colonies of diverse sucking insects) that exchange protection for nutritious secretions and excretions [34]. Thus, the likelihood that the partner will benefit from consistent attendance by protectors will increase.

In a wide range of species, individuals observe conspecifics to learn how to find and handle mutualistic partners or the commodities they offer. If these conspecific individuals are members of a family group or are closely related, both are likely to benefit from this shared information—the observer directly and the observed through indirect inclusive fitness benefits. When they are unrelated or of different species, such information sharing could still come about through direct reciprocity or fitness interdependence (sharing information in some situations can also be costly to the individual being eavesdropped upon [35], a situation we do not consider here). Bumblebees, for example, are well documented to observe and thereby learn how to handle complex flowers from other individuals—a time-consuming process but one that increases handling proficiency and colony-level food intake [36]. Moving away from social insects, certain bats learn the location and quality of fruits from others, including their mothers [37], and seed-dispersing birds follow other members of their flock to newly located fruit crops [38]. Juvenile cleaner fish learn which client species to associate with by observing adults [39].

The benefits and costs of using social information have been extensively explored (e.g. [40,41]). For example, it is known that not all individuals are equally useful as informers, and there is likely to be selectivity based on traits such as age, experience and familiarity [42]. Our point here is that when such information is employed to locate mutualistic commodities, its use has consequences for both partner species as well as for the cooperative interaction between them. In the most straightforward cases, this is owing to an increase in the predictability that the association will form at all, the speed at which it is formed and/or an increase in the number of partners that will be attracted. Other effects can also be identified. For example, sharing information about floral nectar increases bees’ floral constancy (i.e. their consistency in visiting a given plant species), which in turn increases the likelihood that conspecific pollen will be transferred among flowers, to the plants’ benefit. Models suggest that increased floral constancy resulting from information transfer increases colony fitness as well [43].

Within-species information transfer does not always benefit the partner species. The net benefit that mutualists provide is rarely a linearly increasing function: at some point, enough partner individuals have been attracted that the benefits of interspecific cooperation level off [44]. For example, plants and insects can gain complete protection from their natural enemies with a less-than-infinite number of ant defenders. In fact, the relationship between fitness and partner numbers seems often to be curvilinear, with costs of mutualism accumulating faster than its associated benefits, and intermediate numbers of partner individuals thus being optimal [44]. In such cases, the ability of social species to quickly and effectively recruit in large numbers to partner-offered commodities can potentially tip their interaction from mutualism (i.e. with benefits exceeding costs for both species) to antagonism (with benefits exceeding costs for only one of them). In recent years, the context-dependent nature of mutualism has received extensive attention [45]. The effectiveness of information transfer among social species involved in mutualism is one more factor that can contribute to context dependency.

### (b) Management benefits

There is a second way in which the fate of between-species cooperation can depend upon within-species cooperation: when the benefits that one species receives depend on collective and cooperative actions on the part of their partner species. We term these *management benefits*. Management of mutualistic partners is in these cases clearly in the interests of the species performing these actions. In general, it takes place when the mutualistic partner either is large or is a complex, growing, reproducing collective of individuals. Managing a partner species to obtain benefits from it may require simple group action or a division of labour. The critical point is that it takes a coordinated collective to maintain an interaction with a resource, which (in the present case) is a different species that also benefits. Recent work has framed mutualist management as a form of domestication [46]. Whether domestication in general is mutualistic, and in particular whether species always benefit from being domesticated, is an open question [47]. We focus here on cases in which the partner (domesticated) species potentially benefits from being managed.

Management benefits have been documented in a variety of socially cooperative insects, some molluscs, and a few vertebrates, including humans (e.g. [46,48–50]). The most thoroughly understood cases, however, involve ants [51]. Leafcutter ants are particularly well-documented in this regard [52]. These ants cannot survive without specific taxa of fungi that they cultivate in the nest for food. Managing this critical resource involves complex behaviours, including the weeding out of fungal parasites and chemical suppression of pathogens. Ultimately, daughter queens disperse with a pellet of fungus; without it, their new colony has no chance of success. The fungi are largely dependent upon this mode of transmission, and indeed most cannot be found outside of the climate-controlled environment of ant nests (JJ Boomsma 2023, personal communication). Thus, ants and fungi are mutually dependent and have a long history of coevolution.

While we highlight here that ants manage their mutualistic fungi—an activity that can only be accomplished at the colony level—the fungi are simultaneously managing their ants. We return to this type of reciprocal interaction of within-species cooperation and mutualism in §5.

Ants also mutualistically manage plants [51], and, in a much looser but ecologically critical set of interactions, other insects. A wide array of ant species feed upon and share with colony members the nutritious secretions and excretions of butterfly larvae (families Lycaenidae and Riodinidae) and Hemiptera (aphids, scale and treehoppers) [27]. In return, the ants protect these food sources from attack by their own natural enemies. That these associations succeed so well is fundamentally linked to ants’ collective behaviour. It generally takes a group of ants to afford protection to their partner species, particularly those that themselves feed in aggregations (see §4). Some ants carry these partners into the nest, where they gain protection not only from their enemies but also from harsh abiotic conditions (e.g. [53,54]).

## 4. Between-species cooperation affects within-species cooperation

In previous sections, we have laid out how within-species cooperation can influence between-species cooperative interactions. We now consider the reverse: how the existence and success of between-species cooperation can alter within-species cooperation. The difference in the effects discussed in these two sections (§§4a,b) can be subtle. Furthermore, both can occur at the same time in complex feedback loops, a point we return to in §5. However, the phenomena we discuss below are, mechanistically speaking, distinct from those covered above. They also have historically been treated in a different body of literature, one more evolutionary than ecological. These considerations merit the separate coverage we provide.

### (a) Aggregating to obtain benefits from another species

In §3b, we considered cases in which the benefits a species receives from mutualism hinge on being managed by a partner that exhibits within-species cooperation. We now reverse that perspective: within-species cooperation *itself* can be shaped by the need to gain the benefits other species provide. This will generally occur in between-species cooperative interactions that bring together groups of individuals, one of them (our focus here) a species exhibiting within-species cooperation and the other a species that occurs in a group that benefits from being managed. We note that it is possible that both species exhibit within-species cooperation, but for simplicity, we do not consider that case here.

One phenomenon that fits into this category is when a species’ group size influences the benefits it obtains from interacting with another species exhibiting within-species cooperation. Among ant-tended insects, occurring in isolation makes it difficult to attract protectors at all [55]. When this is the case, aggregation will be favoured. Within species that aggregate to obtain mutualistic benefits, larger groups are well documented to attract more partner individuals. For example, ants have been shown to preferentially associate with larger assemblages of treehoppers [56]. It is less clear whether this effect has acted as a selective force on group size [57]. This is because even when larger groups attract more mutualists, per-capita benefits of between-species cooperation appear to generally be smaller in those groups: more ants are attracted, but not in proportion to the group size [58,59]. Other effects also come into play. For example, Morales & Zink [57] have shown that while the probability of treehopper (*Publilia concava*) oviposition increased with ant attendance, it decreased as the treehoppers’ own density increased, suggesting a cost to living in larger groups. As pointed out earlier, the benefits of protection can be highly context dependent, with ants in some cases being outright detrimental. Consequently, it is likely impossible to identify a group size that would predictably maximize the benefits of between-species cooperation.

We note that the attraction of mutualists is only one of many possible causes of group living in species exhibiting within-species cooperation. Aphids aggregate because they are clonal, although the rate of population growth can be keyed in complex ways to the level of ant-tending [34]. With regard to treehoppers, Morales & Zink [57] argue that they feed in groups not because groups experience higher benefits from ant mutualists, but because the presence of those mutualists triggers egg-laying.

Between-species cooperation has also been invoked at times to explain the evolution of parental behaviours. Billick *et al*. [60] showed that the benefits of parental care in the treehopper *Publilia modesta* only accrue in the presence of certain ants, which are attracted to mother–offspring aggregations and protect the offspring from predators. In the absence of ants, parental care actually leads to lower offspring survivorship. Looking across the three families of Hemiptera that constitute the treehoppers, Fletcher [61] found phylogenetic evidence for the repeated evolution of linked parental care and ant-tending: ant attendance, specifically egg guarding, has always preceded the evolution of parental care. Indeed, maternal care has never evolved in treehoppers in the absence of ant attendance. Interestingly, maternal care might have evolved in the context either of raising the benefit that the mutualist provides (drawing ants nearer to eggs, which are in need of protection [61]) or reducing the costs it inflicts (protecting eggs from ant predation [62]).

### (b) Kin recognition facilitated by symbiotic partners

The benefits that mutualistic symbionts confer upon their hosts include the provision of limiting nutrients and protection from the abiotic and biotic environment. The microbes, in turn, benefit from these associations; many have long histories of coevolution with their hosts, with some being transmitted vertically from parent to offspring [63]. Symbioses of this type are ubiquitous in species exhibiting within-species cooperation. Insects and vertebrates exhibiting various degrees of sociality [64,65] have been well studied in the context of their interactions with mutualistic symbionts. Indeed, there have been complex ecological and evolutionary feedbacks between within- and between-species cooperation in some of these systems (e.g. [7]), a point that we develop in §5.

Here, we point to one mechanism by which host–symbiont cooperation can directly influence within-species cooperation in hosts. Symbiotic bacteria are involved in the production of a variety of odours; when members of the same social group harbour similar microbial communities (through mechanisms we discuss in §5), they will smell similar (e.g. [66]). This chemical information can potentially be exploited for kin recognition. For example, it has been proposed that when bacteria ferment protein and lipids in mammalian scent glands, odorous metabolites are produced that can then be co-opted as components of chemical signals [67]. Testing components of this hypothesis in spotted hyenas, Theis *et al*. [64] showed that scent secretions are in fact dense with fermentative bacteria and that bacterial communities are more similar among hyenas belonging to the same clan (social group) than among those from different clans. While it remains to be shown how these group-specific odours are used, this does point to the ability of between-species cooperative interactions—here, specifically, mutualistic symbioses—to mediate social behaviour within animal groups. Kin recognition, as well as mate preferences, have now been linked to microbial symbionts in a variety of insect species as well [68–70].

## 5. Interactive effects and feedbacks interweave within- and between-species cooperation

Above, we have highlighted the effects of within-species cooperation on between-species cooperation and the reverse. In doing so, we adopted a unidirectional approach (i.e. taking the perspective that one form of cooperation affects the other). However, in certain situations, complex feedback is likely between these two levels of cooperation. The most prominent examples involve host species and their beneficial microbes. When mutualisms with microbes are essential to the survival of individuals that live in social groups, and these microbial symbionts live nowhere else, their fates are woven together. In this section, we briefly highlight some of the dynamics that can result.

We have pointed in §4b to the ubiquity of host–symbiont mutualisms in nature. Without certain symbionts, hosts have dramatically reduced survival at best; commonly, they cannot persist at all. Many of these symbionts, in turn, cannot survive outside of their hosts. Although it can be difficult to pinpoint the benefits that symbionts receive, their survival clearly requires a host environment. For this reason, reliable transmission of symbionts between hosts is to the evolutionary advantage of both partner species. Except in the case of internal transmission from mother to offspring, this requires close contact among free-living, conspecific individuals. Not surprisingly, then, symbiont transfer fundamentally affects and is affected by the social behaviour of their hosts.

The best-studied systems involve group-living insects and their mutualistic gut bacteria. There are three especially well-documented mechanisms by which social or gregarious insect hosts acquire and exchange symbionts. First, symbionts may be shed or excreted into the environment, where they are incidentally encountered by offspring or other group members. Environmental transmission of symbionts is an inherently risky strategy because of the lack of assurance that partners upon which survival depends will be found [71,72]. Nevertheless, it is widespread in nature, seen, for instance, in whiteflies and certain true bugs [73,74]. The other two mechanisms, exhibited mostly by social species, carry considerably less risk of failure. Certain eusocial species, notably bees, ants and termites, acquire and share symbionts from conspecifics via trophallaxis, the direct transfer of oral fluids via mouth-to-mouth feeding [75]. Finally, these eusocial species, as well as a wider variety of gregarious ones (including certain bugs, beetles and cockroaches) engage in coprophagy, or the consumption of conspecific faeces immediately after excretion [72,75]. In organisms exhibiting trophallaxis and coprophagy, networks of social interactions provide a channel for the transmission not only of individual symbiotic mutualists but of entire microbiomes, to the point where this microbiome is essentially shared among members of a social group [76]. Shared microbiomes are now well documented in other group-living taxa too, including mammals [64] and birds [77].

Not surprisingly, evolution has shaped the host social structure so as to facilitate symbiont maintenance and transmission. Kin transmission of certain gut bacteria may have been essential in reinforcing sociality in both vertebrate and invertebrate herbivores, many of which have no ability to derive nutrition from the plants they eat without the action of these symbionts [78]. Going even further, arguments have been made that microbe transmission has been a key selective factor in the evolution of eusociality [75]. In termites, vertical transmission of gut symbionts has been shown to have evolved before eusociality did, and it may have been a precondition for eusociality to arise. Termites periodically lose these symbionts, however. This eliminates the option of living independently because it requires that the symbionts be obtained from another individual, which occurs via access to the hindgut fluids, usually but not always of a nest mate [79,80].

A particularly well-documented system in which between-species cooperation has shaped social evolution involves fungus-farming ambrosia beetles (Curculionidae: Scolytinae and Platypodinae). Ambrosia beetles are wood-borers; they and their fungi colonize dead and dying trees, where they are able to feed as the wood decays. In a few lineages, this nutritional mutualism facilitated the evolution of large, long-lived, communal beetle colonies, in which generations overlap and offspring are cared for communally [81]. Farming the fungi, in turn, requires cooperative behaviour on the part of the beetles [50,82]. This socially mediated promotion of beneficial fungi is a form of mutualist management, as discussed in §3.

Thus, there is strong evidence that between-species cooperation—in this case, mutualisms with obligate, symbiotic microbes—has shaped the evolution of within-species cooperation—in this case, in the host species. Is there reciprocal evolution in the symbionts, and on the symbiosis itself? Such evolutionary feedbacks are in fact well-documented [82]. At least, some hosts and vertically transmitted symbionts, for example, have a clear history of coevolution and cospeciation (e.g. [83]). Gene loss and the accumulation of mutations have left certain of these symbionts incapable of life outside a host that cannot survive without them, locking their fates together [84]. Certain traits of such ‘domesticated’ symbionts are best understood as adaptations for increasing host fitness (e.g. [85]). There is evidence that the hosts impose selection on symbiont traits as well. Sociality, and kin selection in particular, has been argued to function so as to punish cheating symbionts, stabilizing and reinforcing mutualism [86,87]. This is expected to be critical even in the most codependent of host–symbiont systems [88].

## 6. Benefits of cooperation that are obtainable from either heterospecifics or conspecifics

So far, we have focused on consumer–resource mutualisms, a form of cooperation that takes place almost exclusively between species occupying different trophic levels. Here, we turn our attention to mutualisms that confer benefits that are, in principle, obtainable from either conspecifics or heterospecifics; partners in such mutualisms almost always occupy the same trophic level. Between-species cooperation in such cases commonly takes place within groups of individuals collectively referred to as *mixed-species groups* (MSGs) [13]. We begin this section by describing the natural history of MSGs. We then show how within-species cooperation, in the form of intraspecific sociality, is often the basis for the formation of MSGs. Finally, we explore how participating in MSGs in turn shapes within-species cooperation. Our aim is not to review the literature on this topic exhaustively (see e.g. [3,13,89]) but to illustrate different ways in which within-species cooperation intersects with MSGs. However, given that a disproportionate amount of MSG research has been on terrestrial mixed-species bird flocks in the context of predator defence, our examples draw more heavily from this system.

### (a) Diversity, prevalence and nature of benefits provided in MSGs

MSGs are moving or stationary groups of multiple species held together by interspecific social interactions [13]. In the case of stationary groups, while a resource (e.g. a basking site or fruiting tree) acts as the primary attractor for individuals to a particular location, interspecific interactions might influence which among equally attractive resource locations are chosen, leading to mixed-species grouping. For example, two species of lizards might choose to aggregate at the same basking site for mutual anti-predatory benefits. Unlike the between-species interactions discussed earlier, which never involve more than two species, MSGs range in size from two species (e.g. coyote–badger hunting associations [90] and primate mixed-troops [91]) to 60–70 species in some terrestrial bird communities (e.g. [92]). Species participate in MSGs as single individuals, mated pairs, family groups or larger groups containing kin and non-kin. Correspondingly, the total number of individuals in MSGs also ranges widely, from only two individuals in the coyote–badger association to hundreds of individuals in some mixed-species bird flocks and fish schools. For the purposes of this article, we treat MSGs as cases of between-species cooperation, i.e. it is reasonable to assume that all participants benefit by participation in MSGs, given that they continue to remain in them. Cooperative benefits in the case of MSGs are usually in the form of fitness interdependence [93,94] and need not involve kinship or reciprocity. At the same time, we note that individual pairwise interactions in MSGs, few of which have been quantified to date, can, in principle, be mutualistic, commensal, or even parasitic. By the same reasoning, we treat all intraspecific social groups as cases of within-species cooperation.

MSGs have been hypothesised to provide a variety of benefits, including protection against predators and improved foraging, thermoregulation and reproduction [13]. Broadly, these benefits are of two types: access to resources such as food, mates and nesting locations, and the amelioration of environmental stressors such as predators, pathogens, extreme temperatures and desiccation. Each benefit can be obtained through either of two mechanisms: via the exchange of social information or through coordinated activity. Sridhar & Guttal [3] identified 150 unique combinations of higher-order taxonomic groups (order and above) and behaviours in which such benefits are obtained through the formation of MSGs.

### (b) The role of within-species cooperation in MSGs

MSGs are an arena in which within-species and between-species cooperation clearly intersect. First, species that show cooperation within species are ubiquitous members of MSGs. Using data on sizes and richness of MSGs in different taxa compiled by Goodale *et al*. [13], a crude calculation of individuals per species indicates that MSGs almost always include at least one species whose intraspecific group size ≥3. This is true also of terrestrial mixed-species flocks of birds, the best studied MSG system to date (based on Zou *et al*.’s [95] global compilation of flock richness and sizes). Furthermore, in taxa such as arthropods [96] and mammals [97], MSGs are only observed in taxonomic groups characterized by strong intraspecific sociality (with the exception of the coyote–badger association [90]).

Species that show within-species cooperation are not only ubiquitous in MSGs but crucial for their formation. Sridhar and Shanker [98] found, through observations of bird MSGs at the initial stages of formation, that groups are joined by new species only if they contain an intraspecifically social species. Playback experiments in bird MSGs have demonstrated that the vocalizations of intraspecifically social species are particularly effective in attracting other species (e.g. [99,100]) and experimental removal of such species leads to a reduction in MSG activity [101]. Finally, regional [102] and global [103] comparative analyses suggest that intraspecifically social species, and among them cooperative breeders in particular [104], likely play key roles in the formation of MSGs. As in bird MSGs, participation of intraspecifically social species in MSGs in other taxa such as fish [105,106] and mammals [97] suggests similar dynamics.

Why is within-species cooperation important for the formation of MSGs? As mentioned earlier, the specific cooperative benefits obtained from MSG forms are also exchanged among conspecifics in many species. Species that are unable to form conspecific social groups to obtain such benefits, either because of strong intraspecific competition [107–109] or because enough conspecifics for group formation are unavailable locally [3], obtain the same by associating with species that cooperate intraspecifically. Such association is probably also facilitated by the predisposition (or at least tolerance) to social interactions in intraspecifically cooperative species [5]. Below, we describe three broad classes of benefits that are obtainable through such associations.

#### (i) Social information

Intraspecifically solitary species benefit from social information about food (e.g. [110]) and predators [111–114] from intraspecifically social species in MSGs. In bird MSGs, Paridae members are thought to play the role of ‘keystone information providers’ [115] or ‘community informants’ [116]. It is likely that other bird families characterized by strong intraspecific sociality, such as fulvettas (e.g. [117,118]) and thornbills (e.g. [119]), also play similar roles. Such species are known to vocalize information about food and threats for the benefit of conspecifics in MSGs, which is eavesdropped upon by solitary heterospecifics as well as other intraspecifically social heterospecifics; occasionally, parids are also known to actively direct information towards heterospecifics (e.g [110]; the benefits that intraspecifically social species might gain from MSGs are discussed in §6c).

#### (ii) Benefits related to group size or coordinated activity

Even when no information is exchanged, associating with intraspecifically social species can provide benefits of coordinated activity to intraspecifically solitary species. For example, intraspecifically social species have a stronger incentive to perform costly mobbing activity of predators to protect conspecifics from danger [89]. Studies have shown that the tendency for such species to mob increases in the presence of kin (e.g. [120,121]). There is also evidence from comparative analyses that species that live in stable social groups are more likely to mob than those that live solitarily [122]. Functionally important lineages in bird MSGs, such as parids and fulvettas, are known to be among the most prominent mobbers, whose mobbing activity and vocalizations provide anti-predation benefits to intraspecifically solitary species in the vicinity (parids: [123]; fulvettas: [118,124,125]).

Intraspecifically social species also provide group-size-related anti-predatory benefits to intraspecifically solitary species, including encounter dilution [126], confusion of predators [127] and selfish-herd effects [128]. These mechanisms are a form of ‘safety in numbers’ in which the protection afforded is statistical, and need not involve any information transfer.

#### (iii) Cues for the presence of MSGs

Intraspecifically social species might also serve as cues by which other species locate and join MSGs (e.g. [100]). Their large intraspecific group sizes make them visually conspicuous and, in the case of vocal species like primates and birds, their noisiness adds an auditory component, both of which serve as markers for the presence of MSGs.

### (c) Influence of MSGs on within-species cooperation

Above, we examined benefits obtained by intraspecifically solitary species from intraspecifically social species in MSGs. Here, we turn our attention to three benefits that intraspecifically social species might gain from such groups.

#### (i) Augmenting within-species cooperation through MSGs

Intraspecific competition and low availability of conspecifics can set limits on intraspecific group sizes in intraspecifically social species. In such situations, forming MSGs with another intraspecifically social species or individuals of multiple intraspecifically solitary species or both provide a route to augment social group benefits. For example, the white-browed scrubwren (*Sericornis frontalis*) and superb fairy-wren (*Malurus cyaneus*), both intraspecifically social species, occur together in MSGs. Playback experiments have shown that the responses of both species to each other’s warning calls were not different from responses to conspecific calls, suggesting that these species, in addition to information from conspecifics, might also benefit from heterospecific information for protection from predators [129].

#### (ii) Complementing within-species cooperation through MSGs

Intraspecifically social species also engage in MSGs to complement the benefits they obtain from conspecifics. For example, in an MSG of tamarins, Peres [130] found that the saddle-back tamarin (*Saguinus fuscicollis avilapiresi*) was better at detecting threats from the ground and climbing predators, while the moustached tamarin (*Saguinus mystax pileatus*) took the lead in scanning for threats from the sky and the canopy. Among birds, a unique system of complementary exchange involves the family Dicruridae (drongos), whose solitary members form relationships with different intraspecifically social species across the world, including babblers (e.g. [131,132]), sociable weavers [133] and mongooses [134]. In each case, the species of drongo involved gains food, either in the form of prey flushed by the social species’ movement or actively snatched from them; in exchange, the drongo provides vigilance and warning against predators, and therefore, in a sense, frees the social species from the need to post a sentinel of its own. Another context involving complementarity is cooperative hunting. A number of cooperative hunting MSGs involving intraspecifically social seabird, cetacean and fish species have been reported across the oceans of the world, in which the different species play specialized roles in a coordinated fashion to increase the likelihood of prey capture [135,136]. Cetaceans have also been reported to cooperate with humans to hunt fish in at least five locations around the world [22].

#### (iii) Switching to MSGs from within-species cooperation

In species that show fission–fusion social dynamics [137], individuals can switch between associating with only conspecifics, only heterospecifics or both, in a context-dependent fashion. For example, Camacho-Cervantes *et al*. [138] showed experimentally that the Trinidadian guppy, a species known to be invasive in at least 70 countries, shows a preference for conspecifics over heterospecifics but readily associates with heterospecifics when conspecifics are absent. This ability to switch to grouping with heterospecifics, the authors note, might explain the guppy’s invasion success. Similar tendencies have been documented in a variety of invaders, including birds, other fish, lizards, mussels and snails, leading to the hypothesis that invaders associate with heterospecifics to overcome Allee effects during the early stages of invasion [139]. Another situation in which a natural preference for conspecifics might be overridden is in relation to familiarity. Ward *et al*. [140] found that chub (Cyprinidae) naturally preferred conspecifics over heterospecifics as social partners, but when presented with familiar heterospecifics and unfamiliar conspecifics, they chose the former. Association with familiar shoal mates might be for greater shoal cohesion, reduced aggression from shoal mates and greater social learning.

## 7. Discussion

Cooperation among individuals generates social networks that structure the ecology, evolution and behaviour of species ranging from humans to bacteria. Cooperation also takes place across species, often ones that occupy different trophic levels and in which consumption is a key element. Our central point here is that these two levels of cooperation often take place simultaneously and can facilitate each other. These linkages, which we have summarized in figure 1, have the potential to weave together social networks and trophic dynamics, contributing to the structure and functioning of ecological communities. Below, we first summarize the ways in which the two levels of cooperation interact. We then highlight two features likely to determine the ecological and evolutionary dynamics of these cross-linkages. We next consider why, given their likely high frequency in nature and biological significance, we do not know more about these phenomena. Finally, we conclude with questions for further investigation, explicitly focusing on the larger- and longer-scale effects of interactions among forms of cooperation.

We have laid out two general ways in which within- and across-species cooperation are cross-linked. First, cooperation of one type can change the context in which cooperation of the other type functions and thus potentially its outcome. We have delineated and offered examples of three possibilities: (i) within-species cooperation modulates benefits for a heterospecific partner; (ii) between-species cooperation affects the dynamics of within-species cooperation; and (iii) both of these processes take place interactively. A second type of interweaving emerges when the goods or services that cooperation makes available can be obtained either from members of the same or different species. This brings cooperation at the two levels into direct interaction, forging complex and dynamic linkages between them.

We offer two general observations about the interactions between within- and between-species cooperation. The first is that these effects are highly context-dependent: their strength and sometimes their direction vary with ecological setting. In recent years, between-species cooperation (mutualism) has been extensively documented to show a high degree of context dependency [45]. A consideration of the benefits and costs accruing to partners suggests why this is the case. Organisms use mutualists to gain resources that they lack or that are difficult to acquire. As a rule, on each side, there is some cost involved in fostering relationships with species that can provide these resources. Thus, the net effects of mutualism (its benefits minus its costs) are higher, for example, where the resource that mutualists can provide is more essential, where that resource is unavailable elsewhere, or where the costs of providing a resource in return are lower. Although considerably less studied, the benefits of within-species cooperation can be context-dependent as well (e.g. [141]). In combination, then, the outcome of the within- and between-species cooperation link will be highly dependent upon fluctuating local conditions. As one example, it has been documented repeatedly that certain insects suffer reduced survival in the absence of their ant protectors [142]. As we have discussed (§3a), a key to this interaction is ants’ collective behaviour, including an efficient within-nest communication network that permits rapid recruitment to food sources. However, these collective actions do not always benefit their partners. Once ant-tended species have attracted enough partners to gain full protection, recruitment of yet more ants is unhelpful, and in some cases detrimental [143]. Indeed, especially when ant-tended species are not under threat, the net effect of ants can shift to being mildly or even massively costly [144]. Similarly, in MSGs, we observe complex fission–fusion dynamics as a function of the presence and abundance of predators, food and potential social partners [3]. It seems likely that the resulting variation in outcome from within- and between-species cooperation linkages reduces the ability of natural selection to shape these interactions. The evolutionary implications of context dependency in general have been minimally studied (but see [145–147]), making it difficult at present to generate predictions.

A second observation is that the community-level consequences of the interaction of within- and between-species cooperation should depend on whether the between-species cooperators occupy the same or different trophic levels. As we have discussed in §2, as a rule, mutualists that occupy different trophic levels interact via consumer–resource interactions, exchanging different commodities (e.g. food for protection or food for transportation). In these cases, between- and within-species cooperation weave together trophic levels, with effects on community dynamics that have barely begun to be explored. In contrast, mutualists that occupy the same trophic level (such as those discussed in §6) commonly exchange commodities that are also obtainable from members of the same species. In these cases, linkages are also forged and strengthened within communities but at single trophic levels.

We note that both types of community linkages interact with, and are likely altered by, interactions extending well beyond cooperation. In §3, we pointed to cases in which the presence of a third species (a natural enemy) alters the outcome of the within-and between-species linkage in ant protection interactions. The same effect is seen in MSGs when the benefit of cooperation is protection from predators (§4). Cooperation can confer an advantage in competitive interactions [148,149]; conversely, cooperators occupying the same trophic level may compete fiercely with each other (e.g. [150]). A fuller consideration of the community dynamics of cross-linked cooperative interactions would need to take into account such phenomena.

We have discussed a wide range of species in which within- and between-species cooperation interact. Yet, we are convinced that these interactions are far more common than we have documented and that there are more types of cross-linkages than we have documented here. Why might they have eluded attention?

We believe that our knowledge has been biased by the different approaches, focal questions and systems used when studying these phenomena. Much of the empirically oriented within-species literature focuses on food acquisition as a factor favouring the evolution of cooperation, without explicit attention to the fact that ‘food’ is often another species (sometimes, a mutualist) with its own ecological and evolutionary dynamics. Conversely, the mutualism literature often treats partner species as collectives of identical individuals, glossing over within-guild cooperative and competitive dynamics [151]. More generally, the study of these two forms of interaction has emerged from different subfields of biology: within-species cooperation from animal behaviour and between-species cooperation from population and community ecology [2]. Our point here is that a wider perspective can reveal new and surprising insights relevant to each level of cooperation separately, not only to their interaction. Furthermore, we note that some kinds of interactions between forms of cooperation are well-investigated in some systems but have simply never been looked at in others. In particular, the literature on symbiotic mutualisms is exceptionally rich in information on how host–symbiont dynamics affects and is affected by host behaviour, including sociality. The questions being addressed about these interactions (e.g. how within-species cooperation facilitates the dispersal of mutualists) might profitably be applied to very different types of systems.

We conclude by suggesting five questions deserving of exploration via empirical and theoretical studies. Investigations of these phenomena should lead to a richer appreciation of the cross-linkages among forms of cooperation, as well as a deeper understanding of their roles in structuring communities.

— Does mutualism favour the evolution of sociality, and/or the reverse? More generally, does cooperation at one level set the conditions for its emergence at the other level, and if so, which direction is most prevalent?
— Do traits that facilitate within- and between-species cooperation coevolve?
— Are mutualisms more common within social than within non-social species? If so, why?
— What is the role of between-species cooperation in niche construction? Does between-species cooperation expand the niches of species that exhibit within-species cooperation? Conversely, do mutualisms with cooperative species expand the niches of their non-social partners?
— Do cross-linkages among forms of cooperation help stabilize ecological communities, or otherwise change coexistence conditions?

## Data Availability

This article has no additional data.
